# A multi-attribute approach to evaluating the impact of biostimulants on crop performance

**DOI:** 10.3389/fpls.2023.1214112

**Published:** 2023-08-10

**Authors:** Rodrigo Mendes, Inácio de Barros, Paulo Antônio D’Andréa, Maria Stefânia Cruanhes D’Andréa-Kühl, Geraldo Stachetti Rodrigues

**Affiliations:** ^1^ Embrapa Meio Ambiente, Jaguariúna, SP, Brazil; ^2^ Embrapa Gado de Leite, Juiz de Fora, MG, Brazil; ^3^ Microgeo Biotecnologia Agrícola, Limeira, SP, Brazil

**Keywords:** impact assessment, multi-attribute indicators, rhizosphere microbiome, soil microbiome, sustainable agriculture

## Abstract

An ever-growing collection of commercial biostimulants is becoming available in a wide variety of forms and compositions to improve crop performance. Given the intricate nature of deciphering the underlying mechanisms of commercial products, which typically comprise various biological components, it is crucial for research in this area to have robust tools to demonstrate their effectiveness in field trials. Here, we took a multi-attribute approach to evaluating the impact of biostimulants on crop performance. First, we assessed the impact of a biostimulant on the soil and rhizosphere microbiomes associated to crops in eight reference farms, including corn (3 farms), soybean (2), cotton (2) and sugarcane (1), in different biomes and production contexts in Brazil and Paraguay. Second, we modeled a set of integrated indicators to measure crop responses to biostimulant application, including five analytical themes as follows: i) crop development and production (9 indicators), ii) soil chemistry (9), iii) soil physics (5), iv) soil biology (6) and v) plant health (10). Amplicon 16S rRNA and ITS sequencing revealed that the use of the biostimulant consistently changes the structure of bacterial and fungal communities associated with the production system for all evaluated crops. In the rhizosphere samples, the most responsive bacterial taxa to biostimulant application were *Prevotella* in cotton; *Prauserella* and *Methylovirgula* in corn; and *Methylocapsa* in sugar cane. The most responsive fungal taxa to biostimulant use were *Arachnomyces* in soybean and cotton; and *Rhizophlyctis* in corn. The proposed integrated indicators yielded highly favorable positive impact indices (averaging at 0.80), indicating that biostimulant-treated fields correlate with better plant development and crop performance. Prominent indices were observed for indicators in four themes: soil biology (average index 0.84), crop production (0.81), soil physics (compaction reduction 0.81), and chemical fertility (0.75). The multi-attribute approach employed in this study offers an effective strategy for assessing the efficacy of biostimulant products across a wide range of crops and production systems.

## Introduction

As defined by Yakhin et al. (2017) a biostimulant is “a formulated product of biological origin that improves plant productivity as a consequence of the novel or emergent properties of the complex of constituents, and not as a sole consequence of the presence of known essential plant nutrients, plant growth regulators, or plant protective compounds.” Considering the complexity to determine the underlying mechanisms of action of commercial products, which normally are constituted of diverse biological sources and obtained thru varied industrial processes, one important focus of the research in this field should be directed to proof the biostimulant efficacy (Yakhin et al., 2017). However, to determine the biostimulants technology efficacy more quantitative assessments on field trials are needed (Li et al., 2022).

The soil application of biostimulants is expected to impact not only plant performance, but also the soil/rhizosphere microbiomes associated with plants (Backer et al., 2018; Nuzzo et al., 2020). Soil and rhizosphere microbiomes function as extensions of the plant genome, playing a critical role on plant development and protection (Berendsen et al., 2012; Mendes et al., 2013). Microbial inoculants can modify the native soil community composition and structure, potentially altering soil functioning through changes in the soil microbiome (Mawarda et al., 2020). Microbiome modulation through microbial inoculants represents a sound strategy to promote plant development (Berg et al., 2021). Therefore, understanding the impact of biostimulants on microbial communities associated to crop field conditions is essential to assess their efficacy.

Biostimulants have been used in a wide variety of crops, in a whole range of cropping intensification levels, as well as in diverse agricultural production environments. Many studies have brought significant advances in the knowledge of soil biological functioning and the specific roles of different soil fertility attributes, characteristic to the varied types of soils, forms of management, and environmental contexts (Hungria et al., 2009; Lopes et al., 2013; Chamizo et al., 2018). Sets of biological indicators have also been devised to adequately focus on the role of biostimulants as a special kind of soil quality amendment (Mendes et al., 2015). However, in most instances these soil biology indicator sets apply to a partial assortment of variables, generally restricted to microbial activity, enzymatic functions, and soil organic matter composition (de Faria et al., 2021), lacking consideration on crucial aspects related to environmental, economic, and agronomic endpoints, essential for crop management decision-making.

More recent approaches address comprehensive soil health measures, relying on cutting-edge data analyses that include the use of microbiome machine learning for assessing soil health (Chang et al., 2017; Wilhelm et al., 2022). Nonetheless, such approaches may not suffice when a whole crop performance scenario is sought out, as to provide agricultural management recommendations in real farm settings (van Es and Karlen, 2019; Williams et al., 2020). In this sense, comprehensive indicator sets which aggregate crop performance (i.e., above and below-ground plant vigor, stand status, produce quality, productivity, and revenue), soil physicochemical and biological properties, and plant health markers are needed to properly assess the impacts of biostimulant technology and its role toward the sustainability of cropping systems (Doran, 2002). Given the diversity of formats, measurement units, and expression scales involved in such soil-biostimulant-crop performance impact assessment studies, in relation to the diversity of parameters analyzed, there is relative difficulty in aggregating, interpreting, and expressing the varied set of indicators in integrated indices, which would improve the understanding and communication of performance gains, thus favoring decision-making for adoption and expansion of the technology.

In this study, we first verified the impact of the use of a biostimulant on the microbiome associated to several crop systems, which served as a proxy for biostimulant’s effectiveness. Then, to address the issue of variability and the absence of standardized indicators for biostimulants impact assessment studies, we proposed a multi-attribute system for integrating soil physicochemical, biological, crop performance and health indicators associated with biostimulant technology use. The proposed indicator system aims to favor the registration, interpretation, and communication of integrated impact and technical performance indices, resulting from analyses obtained on-farm. Field assessments were carried out on reference case studies, in cropping systems with a well-documented history of biostimulant application in different crops, distributed throughout a range of productive regions, encompassing an ample variety of soils and climatic conditions.

## Materials and methods

### Selection of commercial biostimulant, reference farms and experimental design

As biostimulant, we selected a well-established commercial product with usage history of over 20 years in South America. The product Microgeo^®^ is a biostimulant applied in a wide variety of crops (Gama et al., 2014; Cardoso et al., 2017; de Almeida et al., 2018; da Silva et al., 2020; Suarez et al., 2020; Filho et al., 2021). This technology is based on a continuous liquid compost and consists of locally adapted microorganisms brewed *in situ* in a field-implemented biofactory, under the influence of the organomineral matrix Microgeo^®^ (patent number PI 0207342-0). The product presents 10^7^ to 10^9^ cells ml^-1^, diversified among fungi, yeasts, and up to 89% bacteria, the main phyla being *Actinomycetota*, *Bacteroidota*, *Cyanobacteriota*, *Bacillota*, and *Proteobacteriota*. We selected eight farms producing corn (3 farms), soybean (2), cotton (2) and sugarcane (1), located in different biomes and production contexts in Brazil and Paraguay, with history of the biostimulant use. Detailed information on location, climate and biome, size of experimental area, history of biostimulant use, planting and sampling dates are described in **Table 1**. In each evaluated production system, the biostimulant was tested against the control, i.e., two treatments – biostimulant *vs* control, in neighboring commercial fields selected as to display as sole contrasting feature the application of the biostimulant. The authorization for soil sampling is registered with the National System for the Management of Genetic Heritage and Associated Traditional Knowledge (SISGen) under number A11C02F.

**Table 1:** Reference farms selected as case studies with a well-documented history of biostimulant adoption, with respective crops, general aspects, biostimulant usage, and sampling information.

Farm	Crop (Cultivar)	Location	Coordinates	Climate zone* and Biome	Area**	Years with Biostimulant Application	Sampling Plant Growth Stage	Planting/Sampling Dates
Corn_MG	Corn (P3707VYH)	Pirajuba, Minas Gerais, Brazil	19°50’57.1”S 48°43’00.5”W	Cwa, Tropical savannas and shrublands	10,000 ha	3	Stage R3	Apr 21/Jul 21
Corn_GO	Corn (AG 8480)	Inhumas, Goiás, Brazil	17°19’10.4”S 50°53’06.2”W	Aw, Tropical savannas and shrublands	1,608 ha	8	Stage R2	Feb 21/Jul 21
Corn_MS	Corn (AG 8480 PRO3)	Itaporã, Mato Grosso do Sul, Brazil	22°00’30.4”S 54°46’47.3”W	Cfa, Tropical broadleaf forest	1,145 ha	15	Stage R1	Mar 21/Jul 21
Soy_SC	Soybean (NI)	Modelo, Santa Catarina, Brazil	26°45’12.4”S 53°05’51.7”W	Cfa, Tropical broadleaf forest	400 ha	7	Stage R2	Feb 21/May 21
Soy_PY	Soybean (Monsoy 6211 IPRO)	Santa Fé del Paraná, Alto Paraná, Paraguay	25°10’46.9”S 54°37’50.0”W	Cfa, Tropical broadleaf forest	450 ha	15	Stage R2	Mar 21/Apr-Jun 21
Cotton_MT	Cotton (TMG 44 B2RF)	Campo Novo do Parecis, Mato Grosso, Brazil	13°43’32.5”S 57°55’48.2”W	Aw, Tropical broadleaf forest	3,000 ha	5	Flowering	NI/Aug 21
Cotton_BA	Cotton (NI)	Luiz Eduardo Magalhães, Bahia, Brazil	11°30’19.8”S 45°44’08.2”W	Aw, Tropical dry broadleaf forest	13,500 ha	5	Reproductive	Jan 21/May 21
Cane_SP	Sugar cane (RB92 8064)	Ribeirão Preto, São Paulo, Brazil	20°49’34.3”S 47°26’55.3”W	Cwb, Tropical broadleaf forest	1,050 ha	2	Pre-maturation	Apr 18/Jul 21

* Köppen-Geiger classification.

** Total area treated with the biostimulant.

NI, not informed.

### Soil and rhizosphere microbiome assessment

Soil from crop inter-rows (bulk soil) and rhizosphere were collected from 5 to 20 cm depth. Rhizosphere samples were collected by removing the whole plant root system from soil and gently shaking to remove excess soil from the root system. Then, the root system was placed in plastic bags and vigorously shaken to obtain the soil adhered to the root system, which was used for downstream analyses. Each rhizosphere sampling replicate consisted of a single plant, and the bulk soil sample was collected in the inter-row next to the plant used for rhizosphere sampling. Therefore, two sample types (bulk soil and rhizosphere) and two treatments (biostimulant and control), considering three replicates, were collected across eight production systems (**Table 1**), resulting in 96 independent samples for microbiome assessment.

Soil and rhizosphere DNA isolation was performed using 0.250 g of soil, which were transferred to 2 mL cell lysis tubes containing glass microbeads (provided by the manufacturer). DNA extraction was performed using the DNeasy Powersoil Pro kit (Qiagen, Hilden, Germany), following the manufacturer’s recommendations. The isolated DNA was subjected to electrophoresis on a 0.8% agarose gel for integrity analysis. Purity was evaluated on a NanoDrop1000 spectrophotometer (Thermo Fischer Scientific, Waltham, MA, USA), using the absorbance ratios of 260/280 and 260/230.

For bacterial community analysis, the hypervariable region V4 of the 16S rRNA gene was amplified using the primers 515F (5’-GTGCCAGCMGCCGCGGTAA-3’) and 806R (5’-GGACTACHVGGGTWTCTAAT-3’) (Caporaso et al., 2010). For fungal community analysis, the ITS1-5F (Internal Transcribed Spacer) region of the rRNA gene was amplified using the primers ITS5‐1737F (5’-GGAAGTAAAAGTCGTAACAAGG-3’) and ITS2‐2043R (5’-GCTGCGTTCTTCATCGATGC-3’). After purification of the PCR product with the AMPure XP Beads kit (Beckman Coulter, Life Sciences), Illumina adapters were ligated in a PCR reaction using Nextera XT Index Primer 1 (N7xx) and Nextera XT Index Primer 2 (S5xx). Subsequently, the product of this reaction was purified and quantified using a NanoDrop spectrophotometer for equimolar normalization of the concentration. A pool was assembled and quantified by qPCR for validation and determination of the final concentration using the KAPA Library Quantification kit for Illumina (Roche). High-throughput sequencing of the amplicons was performed on the Illumina MiSeq platform (2 x 250 bp), in 2x250 bp runs.

### Bioinformatics analyses and statistics

The quality of the raw sequences was checked using the program FASTQC v0.11.5 (Andrews, 2010). Sequences originating from the primers were removed using the Cutadapt v4.2 tool (Martin, 2011). Microbiome analysis was performed using the DADA2 v1.24.0 tool (Callahan et al., 2016), including: removal of low-quality reads (phread <20) and noise (denoising), joining of R1 (forward) and R2 (reverse) sequences, removal of chimeras (using the consensus method), and clustering of representative sequences based on amplicon sequence variants (ASVs). Taxonomic classification was then assigned using the SILVA ribosomal RNA gene database version 138.1 (Quast et al., 2013). Analyses were performed in the R statistical environment (v. 4.2.1) (R Development Core Team, 2014). The taxonomic table containing the count was imported along with the metadata file for analysis in the Phyloseq package (McMurdie and Holmes, 2013) of R. Principal coordinates analyses (PCoA) based on the Bray-Curtis (Bray and Curtis, 1957) distance matrices were performed to evaluate divergence between replicates and samples. Sequencing coverage was evaluated by rarefaction analysis. Alpha diversity indices based on the Chao1 richness estimator (Chao, 1984), observed species, and Shannon-Wiener H’ index were calculated by the Phyloseq package of R. Microbial composition was expressed in relative abundance for all taxonomic levels.

The statistical package DESeq2 v1.36.0 (Love et al., 2014) was used to identify differentially abundant microbial groups. DESeq2 applies negative binomial distribution analyses to evaluate differences by comparing two samples in triplicate. A p-value of <0.05 was used, and heatmaps were generated for visualization of the bacterial and fungal genera that were statistically different between treated and untreated (control) samples.

### Crop development, chemical, physical, and biological analyses

**Figure 1:**
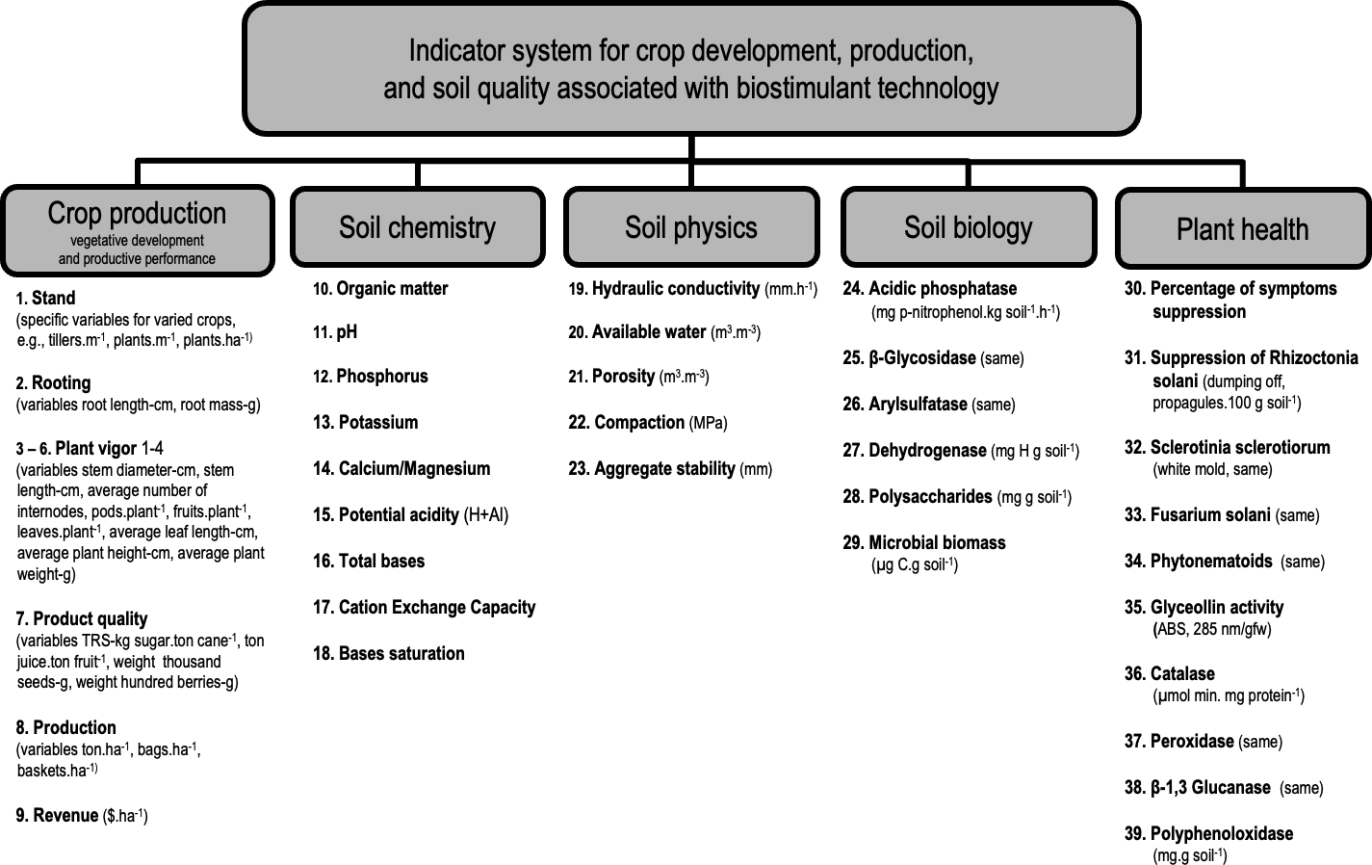
Structure of analytical themes and indicators of crop development, production, and soil quality associated with the adoption of biostimulant technology.

Sampling procedures for crop development and plant biometry were standardized according to the variables appropriate for the different cropping systems (**Figure 1**) and are presented here only as related to the four crop species studied. Sampling was conducted at the specific plant developmental stage as indicated in **Table 1**. For annuals (corn, soybean, cotton) stand quality was assessed by counting plants in five meter transects with three repetitions per treatment, 30-60 days after emergence (DAE). Perennial sugar-cane stand was assessed by counting tillers in 10 m transects with five repetitions per treatment, 120 DAE. Plant vigor indicators (1-4) were estimated by measuring/counting leaves/stems/internodes/fruits/pods in ten randomly selected plants per treatment. Rooting was checked in 10 plants per treatment for annuals, 30-60 DAE; and for sugar-cane through 50x50 cm trenches (deeper when equipment available) in three repetitions per treatment. Product quality (according to appropriate crop variables, **Figure 1**), production and revenue data were obtained from farm managers’ administrative records. Soil samples for chemical determinations were obtained just postharvest, from five 0-20 cm depth subsamples taken from the cropping lines, combined into one sample per treatment. Soil samples obtained from 0-10 cm depth were used for microbiome analysis and for enzymatic activity determinations, always observing medium soil humidity. Nutrients, organic matter, and enzymatic activity determinations were carried out in the same certified commercial laboratory (Laborsolo – Londrina, PR), conforming one single purchasing order (simultaneous). Soil compaction was determined with a penetrometer up to 40 cm depth, in five repetitions per treatment, postharvest. Bulk soil and rhizosphere samples used for microbiome analyses were taken adjacent to the sampling spots used for chemical and biological analyses.

For a better understanding of the correlations between the different indicators and how they are related to both impact values and technical performance of biostimulant technology, we conducted principal component analyses for all variables (indicators) and observations (case studies). The Kaiser criterion was used to select the principal components to retain (Kaiser, 1958).

### Crop system parameters and indicator system

The system of indicators for crop development, soil physicochemical and biological quality, and plant health associated with the adoption of biostimulant technology was structured according to the multi-attribute conception of the APOIA-NovoRural method (Rodrigues and Campanhola, 2003; Rodrigues et al., 2010), according to which the analytical variables obtained in the field are expressed in a utility scale (i.e., indices 0-1, baseline modeled at 0.7). The indicators are integrated into five analytical themes, namely: i. crop production (i.e., vegetative development and productive performance, nine indicators), ii. soil chemistry (nine indicators), iii. soil physics (five indicators), iv. Soil biology (six indicators) and v. plant health (10 indicators, not assessed in the present study, **Figure 1**). The selection of analytical themes and associated indicators to specifically address biostimulant impacts and effects on crop performance departed from a literature review of research previously carried out on the studied biostimulant (Gama et al., 2014; Cardoso et al., 2017; de Almeida et al., 2018; da Silva et al., 2020; Suarez et al., 2020; Filho et al., 2021), and complemented by Embrapa’s team institutional experience on the subject (Hungria et al., 2009; Lopes et al., 2013; Mendes et al., 2013; Mendes et al., 2015; Mendes et al., 2018; de Faria et al., 2021).

**Figure 2:**
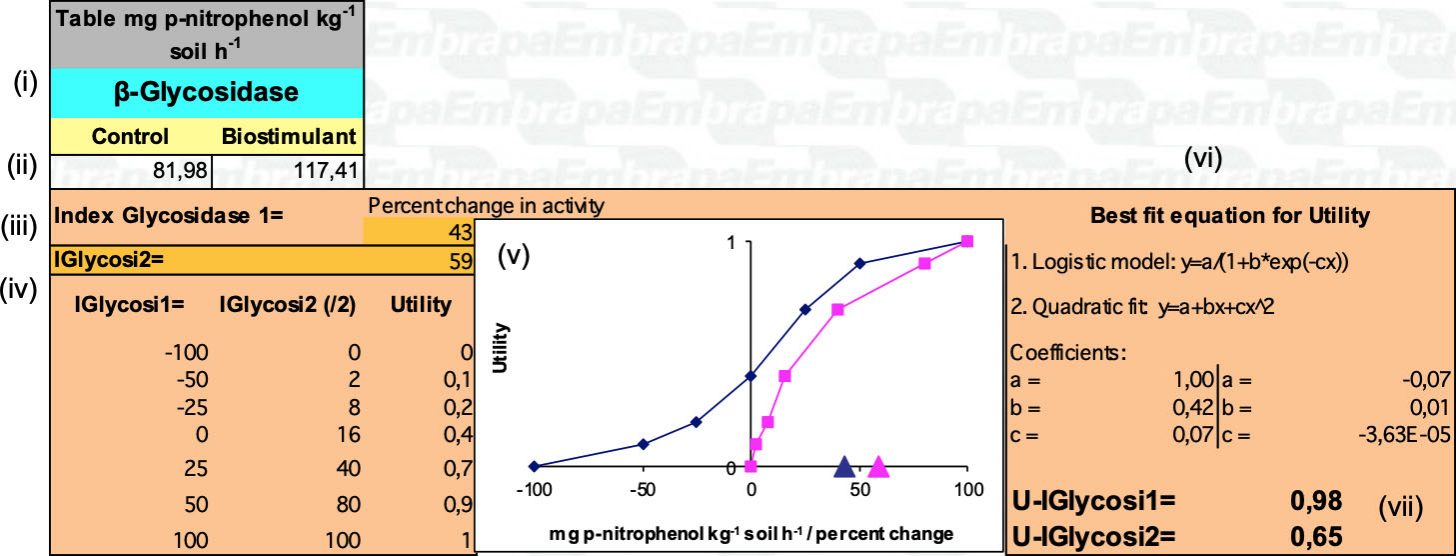
Example of a scaling checklist showing the indicator related to the β-Glycosidase enzyme, from the APOIA-Biostimulant system. The scaling checklists bring (i) the statement of the analytical variable and corresponding indicator (i.e., β-Glycosidase); (ii) cells for data entry of control and treatment samples (biostimulant); (iii) calculated values of the indicators, i.e., Glycosidase 1 index (percentage change in enzyme activity, control x biostimulant) and Glycosidase 2 index (IGlycosi2, enzymatic activity level in the treatment); (iv) correspondence table between the calculated indices (i.e., % change and enzymatic activity) and utility values (scale 0 to 1); (v) graphic expression of these correspondences, with calculated indices markers illustrated on the abscissa; (vi) best fit equations and coefficients for converting calculated indices into (vii) utility values (in this case, U-IGlycosi1 = 0.98; U-IGlycosi2 = 0.65). For additional details on the indicator system construction see Rodrigues et al., 2010.

Information to resolve the indicators is obtained in field assessments, plant biometry estimations, physicochemical and biological analyses of soil samples. Analytical results are entered directly into scaling checklists designed to automatically weight the data and express the impact and technical performance indices for the indicators (**Figure 2**). The integrated indices are then graphically expressed for the considered analytical themes, respective to the local management conditions and productive contexts observed in the studied farms.

The scaling checklists present variable construction for each indicator, always including reference data from the control plots compared to those observed where biostimulant technology is adopted. Calculated impact values (i.e., control *vs* treatment variation) and technical performance (i.e., observed condition *vs* targeted technical standards) are associated with correspondence tables for the utility scale (0 to 1), so that different indicators have their implications properly evaluated, according to specific quantitative variables presented graphically. These matching values are then performed by best fit equations and respective coefficients, for automatic expression of impact and technical performance indices (**Figure 2**).

The composition of the correspondence curves between indicators and utility values is based on probability and sensitivity tests, case by case for each indicator (Girardin et al., 1999). In the probability test, the thresholds of the indicator’s explanatory variable (in **Figure 2**, 0 to 200 mg p-nitrophenol.kg soil^-1^.h^-1^) and its direction (whether positive or negative) are defined in relation to its technical agronomic significance. In the sensitivity test, the value relationship between the indicator’s observed amplitude and the impact/technical performance is defined, according to the correspondence between the occurrence and a standard of technical adequacy (baseline) established in the literature or experimentally.

**Figure 3:**
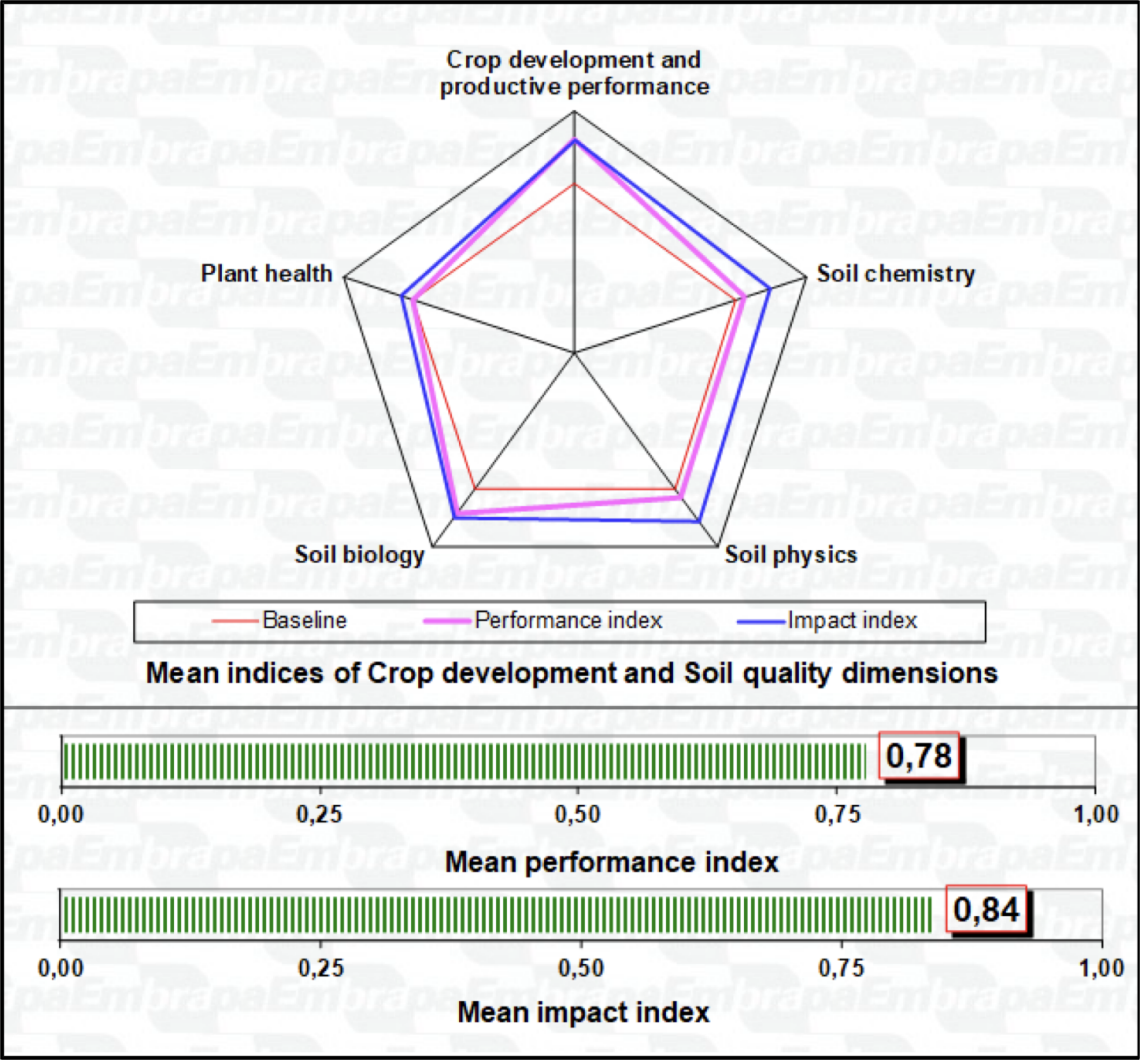
Example of expression of the APOIA-Biostimulant indicator system, showing the baseline (0.7 in red), the impact indices (in blue), and the technical performance indices (in magenta) for each component analytical theme associated with the adoption of biostimulant technology.

The compliance value for the indicators’ baseline is always modeled at 0.7, which corresponds to the situation of stability (zero change, i.e., no impact) or technical suitability for the indicator, according to agronomic standards or benchmarks of productive performance. The evaluation results obtained in the scaling checklists are aggregated by the average value of the utility indices for the set of indicators in each analytical theme and expressed in a summary chart of impact and technical performances. **Figure 3** shows the baseline, the impact indices, and the technical performance indices for each component theme. Additionally, the average indices of impact and technical performance, for the whole set of indicators, are shown in the bars below. From the graph, one can verify the analytical themes that deserve attention for management improvements and those that best represent the impacts and the technical performance achieved, in the specific conditions observed in the studied farms. Specific graphs for each theme present each of the analyzed indicators, allowing the proposition of management recommendations and adoption of practices to promote soil quality and crop development.

The data set for reporting on crop development, soil physicochemical and biological quality, and plant health associated with biostimulant use, as carried out in reference farms, is presented in an Excel^®^ file (**Supplementary Material**) consisting of eight worksheets: Worksheet 1, Reference: presents an explanatory summary of the methodological basis, general aspects and the main bibliographical references, with examples of the applicability of the indicator checklists, in addition to references for institutional contacts. Worksheet 2, Identification: data for the identification of the studied farm, the scale and organization of productive activities, and the space-time context defined for the field observations, selection of samples, and considerations on the objectives of the producer interested in the analyses. The following six worksheets refer to the 39 indicators’ scaling checklists for the five analytical themes (**Figure 1**) and a results worksheet with respective graphic representations (**Figure 3**).

## Results

### Impact of biostimulant use on soil and rhizosphere microbiomes

**Figure 4:**
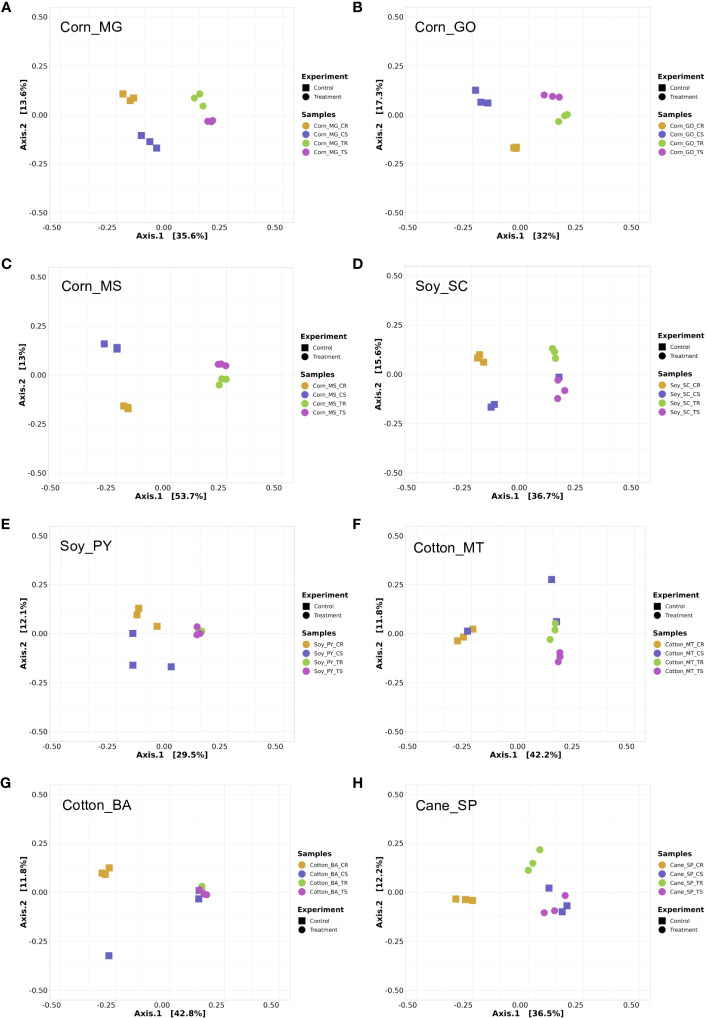
Principal Coordinates Analysis (PCoA) of amplicon 16S rRNA sequencing data based on Bray-Curtis distance matrix. Each data point represents a sample, and the sample source with different colors and shapes are indicated in the figure. Each graph shows four treatments (with 3 replicates), control rhizosphere (CR), control soil (CS), treatment rhizosphere (TR) and treatment soil (TS) for all crop systems evaluated, including corn in MG **(A)**, GO **(B)**, MS **(C)**, soy in SC **(D)** and PY **(E)**, cotton in MT **(F)** and BA **(G)**, and sugar cane in SP **(H)**. PCoA was performed using PhyloSeq package on R software.

**Figure 5:**
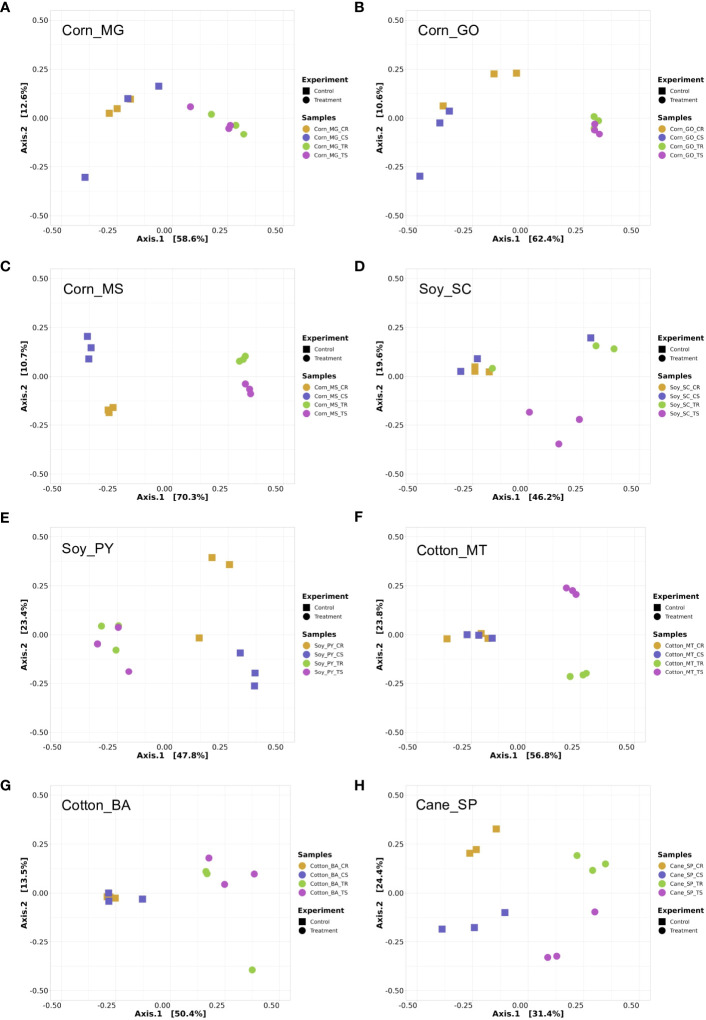
Principal Coordinates Analysis (PCoA) of amplicon ITS sequencing data based on Bray-Curtis distance matrix. Each data point represents a sample, and the sample source with different colors and shapes are indicated in the figure. Each graph shows four treatments (with 3 replicates), control rhizosphere (CR), control soil (CS), treatment rhizosphere (TR) and treatment soil (TS) for all crop systems evaluated, including corn in MG **(A)**, GO **(B)**, MS **(C)**, soy in SC **(D)** and PY **(E)**, cotton in MT **(F)** and BA **(G)**, and sugar cane in SP **(H)**. PCoA was performed using PhyloSeq package on R software.

In general, all locations and crops evaluated revealed a strong rhizosphere effect (**Figures 4**, **5**), i.e., rhizosphere samples cluster apart from soil samples, as the plant exudate is an important driver in the microbiome assembly in the rhizosphere. The alpha diversity observed in all crop systems evaluated did not show significant variation (**Supplementary Figures 1**, **2** for bacterial and fungal communities, respectively). A general composition for bacterial and fungal communities associated with all treatments are shown for each crop system, i.e., corn (**Supplementary Figure 3**), soybean (**Supplementary Figure 4**), cotton (**Supplementary Figure 5**), and sugarcane (**Supplementary Figure 6**). With few exceptions, the beta diversity showed correlation between the structure of microbial communities and the use of the biostimulant, indicating that the use of the technology resulted in change of the microbiome structure in the plant rhizosphere and in the inter-row soil. Bacterial and fungal communities associated with corn showed a different clustering pattern with the biostimulant treatment in comparison with the control treatment for all three fields evaluated (**Figures 4A–C**, **5A–C**). The same pattern was observed for bacterial and fungal communities in soybean, where the biostimulant-treated samples were grouped separated from control samples (**Figures 4D, E**, **5D, E**), except in Soy_SC where one control sample from bacterial community grouped with samples from the biostimulants treatment (**Figure 4D**) and in the fungal community two samples did not cluster as expected (**Figure 5D**). The same general pattern discriminating biostimulant-treated samples was observed for Cotton (**Figures 4F, G**, **5F, G**), except for bacterial communities in soil samples. For sugarcane all treatments were discriminated considering bacterial or fungal communities (**Figures 4H**, **5H**), except bacterial communities in inter-row soil samples, that clustered biostimulant and control samples together (**Figure 4G**).

Further analysis was performed to identify microbial taxa significantly enriched in soil and in the rhizosphere of plants treated with the biostimulant (**Supplementary Figures 7–10**). Significant enrichment or depletion of bulk soil bacteria was found in all fields cultivated with corn and cotton (**Supplementary Figure 7**). Nine bacterial genera were enriched in corn fields where biostimulant was used, including *Longispora* and *Prauserella* (**Supplementary Figure 7**). *Methylovirgula* and *Novosphingobium* bacterial genera were enriched in cotton biostimulant treated fields (**Supplementary Figure 7**). Specific fungal genera significantly changed in abundance in all crop systems evaluated, in total, 37 fungal genera were significantly enriched across different crop systems (**Supplementary Figure 8**). The most biostimulant responsive fungal genera was *Lindtneria* in corn (MS), *Leucocoprinus* in sugar cane (SP) and cotton (BA) and *Clathrus* in soybean (PY) (**Supplementary Figure 8**).

Considering rhizosphere samples, the microbiome analysis showed that the use of the biostimulant enriched or depleted specific bacterial genera in all crop systems, except in Corn_GO (**Supplementary Figure 9**). The most responsive bacterial taxon to biostimulant application was *Prevotella* in cotton (MT), *Prauserella* (MS) and *Methylovirgula* (MG) in corn, and *Methylocapsa* in sugar cane (SP) (**Supplementary Figure 9**). Thirty-one fungal genera were significantly enriched in the rhizosphere across all crop systems (**Supplementary Figure 10**), with the use of the biostimulant. *Arachnomyces* genus was the most responsive genus in soybean (PY and SC) and cotton (MT). The fungal genus *Rhizophlyctis* significantly increased in abundance in corn rhizosphere (MS) in fields treated with biostimulant.

### Impact of biostimulant on crop development, production, and soil quality

All indicators were positively impacted with the biostimulant use (**Supplementary Table 1**). Two sets of interactions among the indicator indices were checked through Principal Component Analysis, one related to the impacts (i.e., relative change from control to biostimulant treatment) and the other relative to the performances (i.e., biostimulant index levels relative to defined technical standards), both relative to soil physicochemical and biological indicators, and to crop development and production. For the biplots of the correlation circle and the observations cloud of the Principal Component Analyses for both impact values and technical performance, refer to the **Supplementary Figure 11**. Among the impact indicators of soil quality, almost all associated to one PC, with significant Pearson’s correlations (α=0.05) for exchangeable cations (K, Ca+Mg, CEC) and, expectedly, the associated variables Total bases and Bases saturation. The enzyme Arylsulfatase related negatively with P in a PC2 and β-Glycosidase stood in a PC3 without being strongly related to any other variable. Regarding the soil performance indices, only CEC, Total bases, and Bases saturation correlated significantly. B-Glycosidase associated positively to pH in PC1 and both correlated negatively with organic matter and Ca+Mg. Arylsulfatase associated negatively with Potential acidity (H+Al).

**Figure 6:**
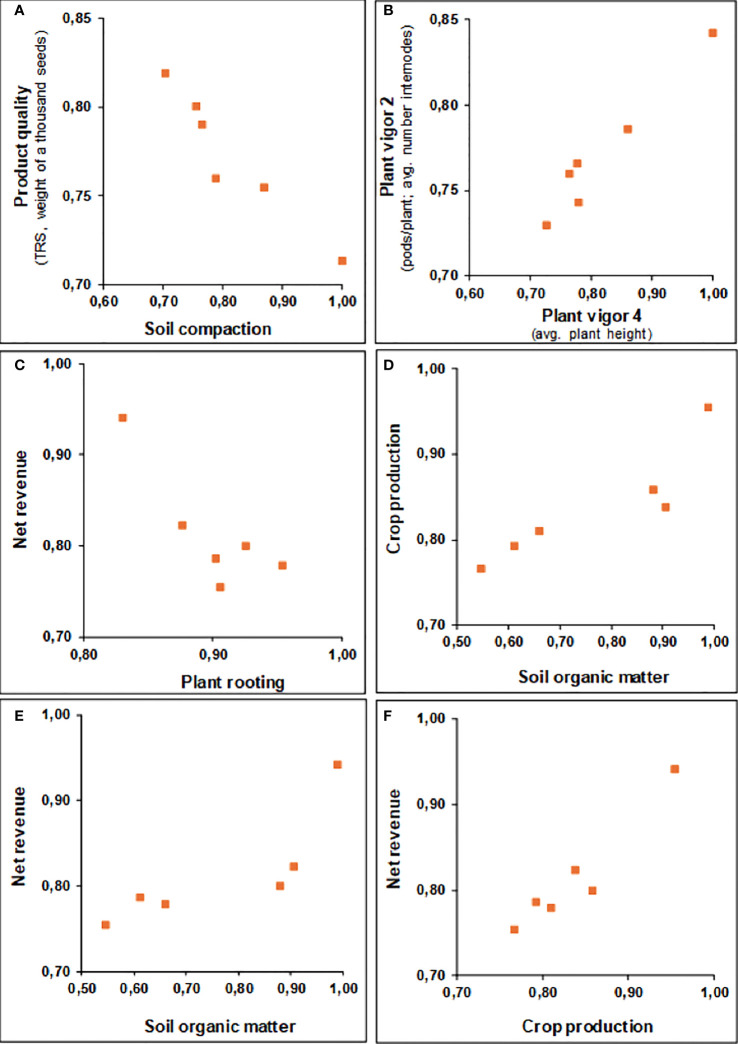
Most meaningful significant Pearson’s matrix correlations (α=0.05) relating multi-attribute impact indices for crop development, production, and soil quality indicators, applied to six reference farms and four different crops (corn, soybean, cotton, sugarcane), comparing control plots against those subjected to biostimulant technology application (varied environmental and temporal contexts). **(A)** Soil compaction *vs* Product quality, **(B)** Plant vigor 4 *vs* Plant vigor 2, **(C)** Plant rooting *vs* Net revenue, **(D)** Soil organic matter *vs* Crop production, **(E)** Soil organic matter *vs* Net revenue, and **(F)** Crop production *vs* Net revenue.

Interesting significant correlations were observed for the crop development and production indicators. The PC1 for the impact indices, which accounts for 40% of the variability (eigenvalue=5.26), strongly associated organic matter, soil compaction (negatively), plant vigor 2 and 3 (related to plant production and biometry), product quality and, expectedly, crop productivity and net revenue (**Figure 6**). Hence, organic matter showed to be relevant in preventing soil compaction and promoting crops development and production. The soil enzymes did not show significant correlation with other variables, with β-Glycosidase associated with plant vigor 4 (average plant height-cm) in a PC3 which accounts for 19.7% of the variability. Interesting negative correlations were observed between the indicator product quality (weight of 1,000 seeds for annuals; TRS for sugarcane) and soil compaction; and rooting with revenue. Regarding the crop development and production performance indices, a PC1 accounting for 38% of the variability (eigenvalue=4.96) equally associated organic matter, plant vigor 1, 2, and 3 (including pods per plant, leaves per plant, average plant height), product quality (weight of 1,000 seeds, TRS), crop productivity and net revenue. The soil enzymes did not show significant correlations, being associated with each other in a PC3, which accounted for 15% of the variability.

## Discussion

Great interest has been directed toward monitoring soil-biostimulant-crop interactions, in order to improve technical and usage recommendations. Most indicator sets assembled, however, lack in scope to properly assess the impacts of the technology on the diversity of cropping systems and farming contexts, as to integrate soil physicochemical and biological properties, plant health, crop performance, and farm results. Using a multi-attribute approach, we demonstrate the positive impact of biostimulant on the microbiome associated to different crop systems and then developed integrated indicators to express these impacts of biostimulants on crop performance and soil quality.

Considering that microbial inoculants and biostimulants are screened and tested in controlled laboratory conditions, it is common to observe lack of consistency when commercial products are tested in field conditions (Kaminsky et al., 2019). After four commercially available microbial amendments failed to promote tomato growth in greenhouse experiments, Nuzzo et al. (2020) suggested that additional confounding variables can interfere in the efficacy of biostimulants evaluated under commercial fields. This fact reinforces the importance of having a reliable strategy to measure biostimulants impact in commercial validation settings, which normally consist in side-by-side comparisons instead of replicated comparisons with proper experimental design. In this sense, the innumerable local variabilities and particularities that influence crop performance, aside of biostimulant usage, are circumvented in the proposed application of the indicator system, by reducing all production environmental complexity to the immediate contrast control *vs* biostimulant treatment, in the several reference farms and crops studied.

A number of studies has demonstrated that inoculants can modify the native soil microbiome directly or indirectly through changes in plant exudates (Trabelsi and Mhamdi, 2013; Mawarda et al., 2020; Cornell et al., 2021). If these changes result in increase of microbial diversity, this could improve ecosystem functioning and consequently plant performance (Bardgett and van der Putten, 2014). In our results, no clear pattern was observed for increased alpha diversity when biostimulant was used. However, all fields treated with biostimulant showed improved plant performance. This suggests that not only increase or decrease of alpha diversity, but also microbial community structure can be correlated with changes in soil microbiome functioning, resulting in better plant development. Therefore, considering the pivotal role of the soil and rhizosphere microbiome for plant development (Mendes et al., 2013) and that the use of inoculants and biostimulants can modify native soil microbiome and consequently alter soil functioning (Mawarda et al., 2020), the assessment of the microbiome as affected by biostimulant use is an important indicator of the biostimulant effectiveness. Despite of diverse soil conditions, in farms located in different biomes and diverse crops, areas treated with biostimulants were discriminated from control areas based on the structure of the microbiome. Although a better mechanistic understanding of the mode of action of complex biostimulants is needed to finely tune their management and measure their effectiveness in field conditions, having a comprehensive set of indicators helps to tackle these challenges.

The integrated analysis of crop development, production, and soil quality associated with the biostimulant use documents the positive impacts and the improvement in technical performance observed in the field trials. Among the main results, it was observed that the impact indices, that is the relative comparison between the controls and the areas with biostimulant, were the most expressive; mainly in the soil biology theme (index 0.84 for arylsulfatase and β-glucosidase enzymes), followed by soil physics (index 0.81 for compaction), and chemistry (index 0.75) and, in response to these positive impacts, the crop production theme (index 0.81). This preponderance of soil biology as the analytical theme of better performance confirms the important effect of those enzymes, as advocated by Mendes et al. (2015; 2018).

The improvements observed in all these analytical themes, in particular the crop production indicators, brought a series of responses of great interest to farmers, including root development (average index 0.89), plant vigor (index 0.83 in length of branches and leaves), vegetative development (index 0.82 in number of internodes, length of stems, pods or grains per plant) and product quality (index 0.77 for protein content in the grains, weight of 1,000 seeds, or TRS–kg of sugar per ton of cane). Most importantly, as an integrated result of these indicators, in areas treated with biostimulant the average productivity was greatly favored in all crops (index 0.84 for bags.ha^-1^ or ton.ha^-1^), resulting in expressive gains in net revenue (index 0.81 for $.ha^-1^ see **Supplementary Table 1**). A close correlation between the productivity and net income indicators (r^2 =^ 0.92), although naturally expected, attests that these gains were achieved without significant cost increases, pointing to the viability of the biostimulant program relative to the rising prices of other inputs, such as conventional chemical fertilizers.

These results of the impact indices (average of cases 0.80 on a scale between 0 and 1), which represent relative gains between treated and control areas, are of great significance, since the performance indices were more modest (cases average 0.69). As these performance indices represent the observed local condition, in relation to appropriate or desired technical benchmarks, it is indicated that there is still room for further gains, as the applications of biostimulant are repeated throughout the harvests, enhancing the expression of the observed impacts. Noteworthy is the fact that even under very contrasting situations, including four different crops in seven distinct ecoregions, soil quality and crop performances were always superior in the areas treated with the biostimulant technology. Also, significant correlations were observed between the averages of the integrated indices of soil quality and the indicators of soil biology performance (r^2 =^ 0.82); followed by the themes soil chemistry and crop production – the latter possibly a consequence of all others.

In conclusion, the microbiome analysis revealed that the biostimulant use consistently impacted the soil and rhizosphere microbiome assembly. The changes in community structure observed in biostimulant-treated fields correlate with better plant development and crop performance. This observation served as a proxy to assess the effectiveness of biostimulants, which was subsequently confirmed through the utilization of the integrated indicators approach. The expression of the results obtained by the use of integrated indicators suggests i) coherence between the system’s analytical themes and indicators with better responses, ii) proper amplitudes of the obtained indices (expressive, but not extreme), and iii) conformity of the thresholds, weighting factors, and graphic scales. These results point to adequate calibration and sensitivity of the set of indicators, for adequately evaluating the impacts of biostimulants on crop performance.

## Data availability statement

The datasets presented in this study can be found in online repositories. The names of the repository/repositories and accession number(s) can be found in the article/**Supplementary Material**.

## Author contributions

RM, GR, and PD’A contributed to conception and design of the study. RM and MD’A-K organized the field work, sampling strategy and data collection. RM performed microbiome analyses. GR and IB developed the integrated indicators model. GR and RM wrote the first draft of the manuscript. All authors contributed to manuscript revision, read, and approved the submitted version.
