# Supplementary material for: A multi-attribute approach to evaluating the impact of biostimulants on crop performance
Source: Front Plant Sci. 2023 Aug 10;14:1214112. doi: 10.3389/fpls.2023.1214112 (PMC10449575; doi:10.3389/fpls.2023.1214112)
Supplement: Supplementary file 2 [file DataSheet_1.pdf]

# Supplementary Material

## A multi-attribute approach to evaluating the impact of biostimulants on crop performance

Rodrigo Mendes[1*], Inácio de Barros[2], Paulo Antônio D'Andréa[3], Maria Stefânia Cruanhes D'Andréa-Kühl[3], Geraldo Stachetti Rodrigues[1]

[1]Embrapa Meio Ambiente, Jaguariúna SP, Brazil; [2]Embrapa Gado de Leite, Juiz de Fora, Brazil; [3]Microgeo Biotecnologia Agrícola, Limeira, Brazil.

*Corresponding author rodrigo.mendes@embrapa.br

**Sequencing data availability**

Amplicon sequencing raw data are available at EMBL-EBI project number PRJEB59860.

# Supplementary Table

**Supplementary Table 1.** Indicator variable values (control *vs.* biostimulant plots) and calculated impact and performance indices related to the assessment dimensions of Crop development, production, and soil quality, obtained in seven reference farms studied in relation to the adoption of plant biostimulant technology. For details on specific variable measurement units, refer to Figure 1.

| Case studies | Corn_MG | | Corn_GO | | Corn_MS | | Soy_SC | | Soy_PY | | Cotton_MT | | Sugarcane_SP | |
|---|---|---|---|---|---|---|---|---|---|---|---|---|---|---|
| Dimensions / indicators | Variable values | Indices | Variable values | Indices | Variable values | Indices | Variable values | Indices | Variable values | Indices | Variable values | Indices | Variable values | Indices |
| Crop development, production | | | | | | | | | | | | | | |
| Stand | 3.7-3.9 | 0.77 | 2.98-3.0 | 0.71 | 3.2-3.3 | 0.74 | 9.7-10.3 | 0.78 | 8.6-9.4 | 0.81 | 7.68-7.73 | 0.71 | 11.6-12.8 | 0.82 |
| Rooting | 12-14 | 0.87 | 280-345 | 0.91 | 12.5-14.0 | 0.83 | 2.63-3.11 | 0.88 | 12-15 | 0.91 | 33.7-41.3 | 0.90 | 25-34 | 0.95 |
| Plant vigor 1 | 318-533 | 0.99 | 16-18.5 | 0.86 | 16.6-19.9 | 0.89 | 7.1-7.3 | 0.74 | 7.3-7.8 | 0.78 | 27.1-30.4 | 0.83 | 2.8-2.81 | 0.71 |
| Plant vigor 2 | 14-16 | 0.85 | 13.3-13.5 | 0.73 | 13.2-15.3 | 0.86 | 36.2-38.6 | 0.78 | 2.6-5.2 | 1.0 | 6.7-7.0 | 0.76 | 16.9-18.0 | 0.78 |
| Plant vigor 3 | 107.4-112.8 | 0.76 | 13.2-14.1 | 0.78 | 74-79 | 0.78 | 14.0-16.4 | 0.87 | 27.4-50.2 | 1.0 | 13.1-14.3 | 0.81 | 1.36-1.38 | 0.72 |
| Plant vigor 4 | 211-235 | 0.82 | 193-197 | 0.73 | 166-178 | 0.79 | 56.7-58.5 | 0.74 | 26.8-30.4 | 0.84 | 47.1-49.3 | 0.76 | 190-200 | 0.77 |
| Product quality | nd | nd | 8.0-8.7 | 0.80 | 296-310 | 0.76 | 135-136 | 0.71 | 182.6-190.4 | 0.75 | nd | nd | 136.3-150.9 | 0.82 |
| Production | nd | nd | 134.7-141.9 | 0.77 | 33-45 | 0.95 | 43.9-49.6 | 0.84 | 11.42-13.2 | 0.86 | 3.17-3.42 | 0.79 | 69.6-76.3 | 0.81 |
| Net revenue | nd | nd | 11719-12214 | 0.75 | 2871-3784 | 0.94 | 7243-8051 | 0.82 | 1884-2047 | 0.80 | 42220-45298 | 0.79 | 7935-8454 | 0.78 |
| Soil chemistry | | | | | | | | | | | | | | |
| Organic Matter | 3.25-3.18 | 0.68 | 3.50-3.10 | 0.55 | 1.60-2.58 | 0.99 | 2.68-3.29 | 0.91 | 3.18-3.78 | 0.88 | 3.21-2.99 | 0.61 | 3.36-3.24 | 0.66 |
| pH impact | nd | nd | 5.2-5.7 | 0.99 | 4.7-5.3 | 0.99 | 5.2-5.4 | 0.99 | 5.2-5.5 | 0.99 | 5.5-5.6 | 0.99 | nd | nd |
| pH perform. | | | | 0.75 | | 0.63 | | 0.66 | | 0.73 | | 0.74 | | |
| P impact | nd | nd | 31.4-17.5 | 0.10 | 132-76.6 | 0.11 | 16.7-19.7 | 0.89 | 15.4-38.1 | 1.0 | 75.4-100.8 | 0.96 | nd | nd |
| P perform. | | | | 0.45 | | 0.99 | | 0.50 | | 0.79 | | 0.83 | | |
| K impact | nd | nd | 2.0-1.7 | 0.46 | 3.3-4.1 | 0.93 | 6.2-7.4 | 0.90 | 5.7-31.3 | 1.0 | 3.3-3.4 | 0.75 | nd | nd |
| K perform. | | | | 0.44 | | 0.83 | | 1.0 | | 0.10 | | 0.74 | | |
| Mg impact | nd | nd | 38.8-20.2 | 0.08 | 2.7-10.6 | 1.0 | 13.0-18.7 | 0.98 | 7.9-22.4 | 1.0 | 23.0-23.7 | 0.75 | nd | nd |

| | | | | | | | | | | | | | | |
|---|---|---|---|---|---|---|---|---|---|---|---|---|---|---|
| Mg perform. | | | | 0.82 | | 0.99 | | 0.82 | | 0.82 | | 0.82 | | |
| H + Al impact | nd | nd | 42.8-50.4 | 0.48 | 57.6-56.3 | 0.72 | 62.5-64.4 | 0.66 | 63.9-55.1 | 0.85 | 57.6-49.3 | 0.86 | nd | nd |
| H + Al perform. | | | | 0.16 | | 0.10 | | 0.02 | | 0.11 | | 0.17 | | |
| Total bases impact | nd | nd | 103.2-65.2 | 0.21 | 17.0-61.3 | 1.0 | 69.3-89.5 | 0.93 | 47.8-135.2 | 1.0 | 77.2-80.1 | 0.75 | nd | nd |
| Total bases perform. | | | | 0.94 | | 0.94 | | 0.97 | | 0.99 | | 0.97 | | |
| CEC impact | nd | nd | 146-115.6 | 0.41 | 74.6-117.6 | 0.99 | 131.8-153.9 | 0.87 | 111.7-190.3 | 0.99 | 134.8-129.4 | 0.65 | nd | nd |
| CEC perform. | | | | 0.99 | | 0.99 | | 0.99 | | 0.99 | | 0.99 | | |
| Bases saturation impact | nd | nd | 70.7-56.4 | 0.41 | 22.8-52.1 | 1.0 | 52.6-58.2 | 0.82 | 42.8-71.0 | 0.99 | 57.3-61.9 | 0.79 | nd | nd |
| Bases saturation perform. | | | | 0.49 | | 0.44 | | 0.51 | | 0.65 | | 0.55 | | |
| <u>Soil physics</u> | | | | | | | | | | | | | | |
| Compaction impact | 2.44-2.18 | 0.82 | 2.65-2.52 | 0.76 | 2.43-2.24 | 0.79 | 3.42-1.78 | 1.0 | 1.88-1.59 | 0.87 | 1.68-1.58 | 0.76 | 2.17-2.16 | 0.68 |
| Compaction performance | | 0.68 | | 0.64 | | 0.67 | | 0.73 | | 0.75 | | 0.75 | | 0.70 |
| <u>Soil biology</u> | | | | | | | | | | | | | | |
| β-Glycosidase impact | 145-131.3 | 0.55 | 123-174 | 0.98 | 26.7-98.8 | 1.0 | 125.1-107.6 | 0.47 | 82.0-117.4 | 0.98 | 81.5-92.7 | 0.86 | 67.7-72.8 | 0.80 |
| β-Glycosidase performance | | 0.71 | | 0.90 | | 0.55 | | 0.60 | | 0.65 | | 0.52 | | 0.40 |
| Arylsulfatase impact | 86.9-130.9 | 0.99 | 70-130 | 1.0 | 6.0-75.2 | 1.0 | 147.0-159.8 | 0.47 | 60.9-85.3 | 0.98 | 57.9-52.2 | 0.54 | 90.9-96.2 | 0.78 |
| Arylsulfatase performance | | 0.90 | | 0.90 | | 0.56 | | 0.60 | | 0.63 | | 0.38 | | 0.70 |

# Supplementary Figures

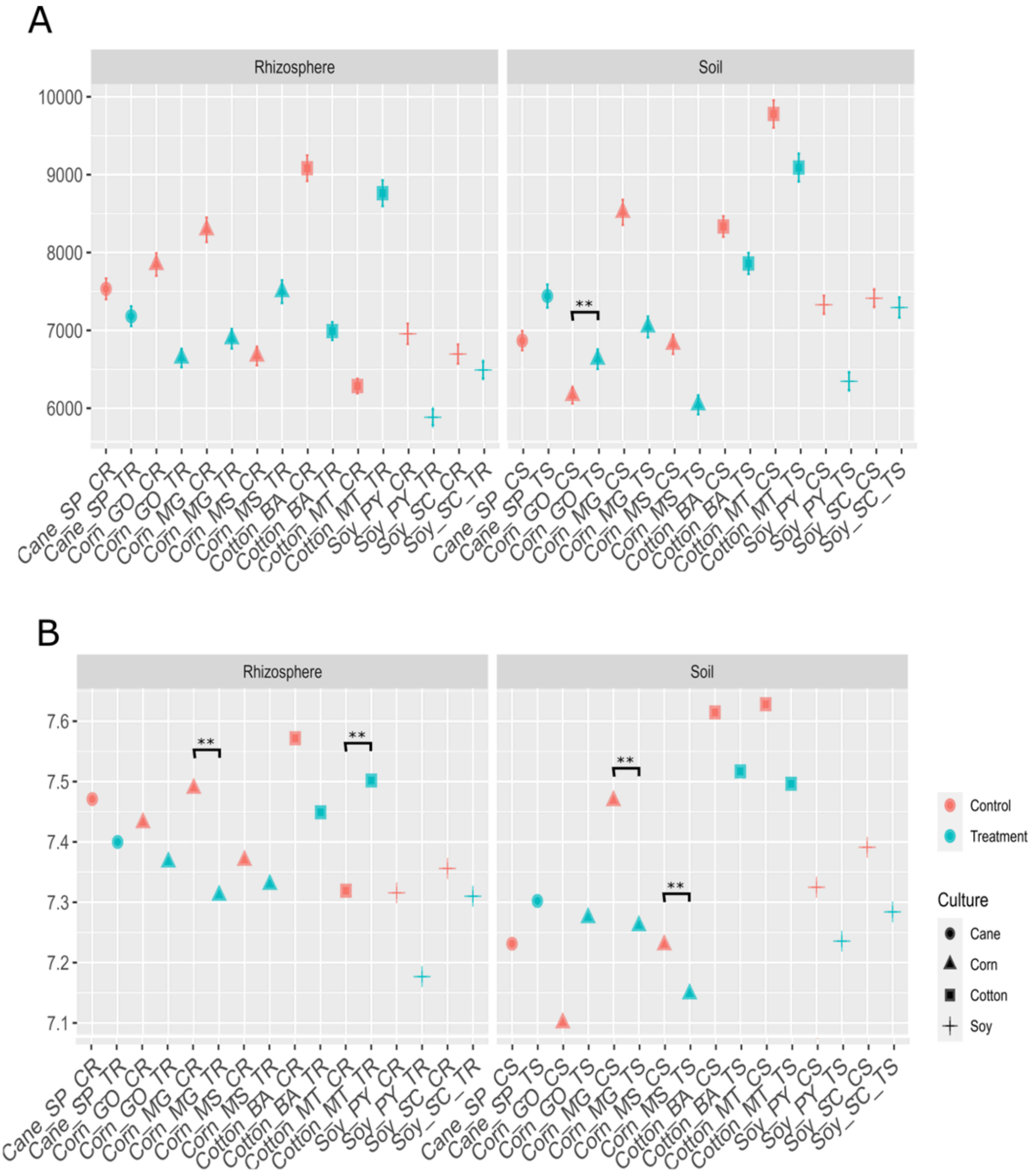


**Supplementary Figure 1.** Alpha diversity analysis of bacterial communities using Chao1 (A) and Shannon (B) indices. The microbiome was analyzed based on their 16S rRNA gene amplicon sequencing. The lines in the graph represent the mean values of Chao1 and Shannon indices for each sample considering 3 replicates. Error bars represent standard deviations. Each crop is represented by a different color as indicated in the key. Locations and all four treatments are indicated, control e(CR) or treatment (TR)Significant differences are indicated by ** (pvalue< 0.05) or *** (pvalue< 0,01) according to T test.

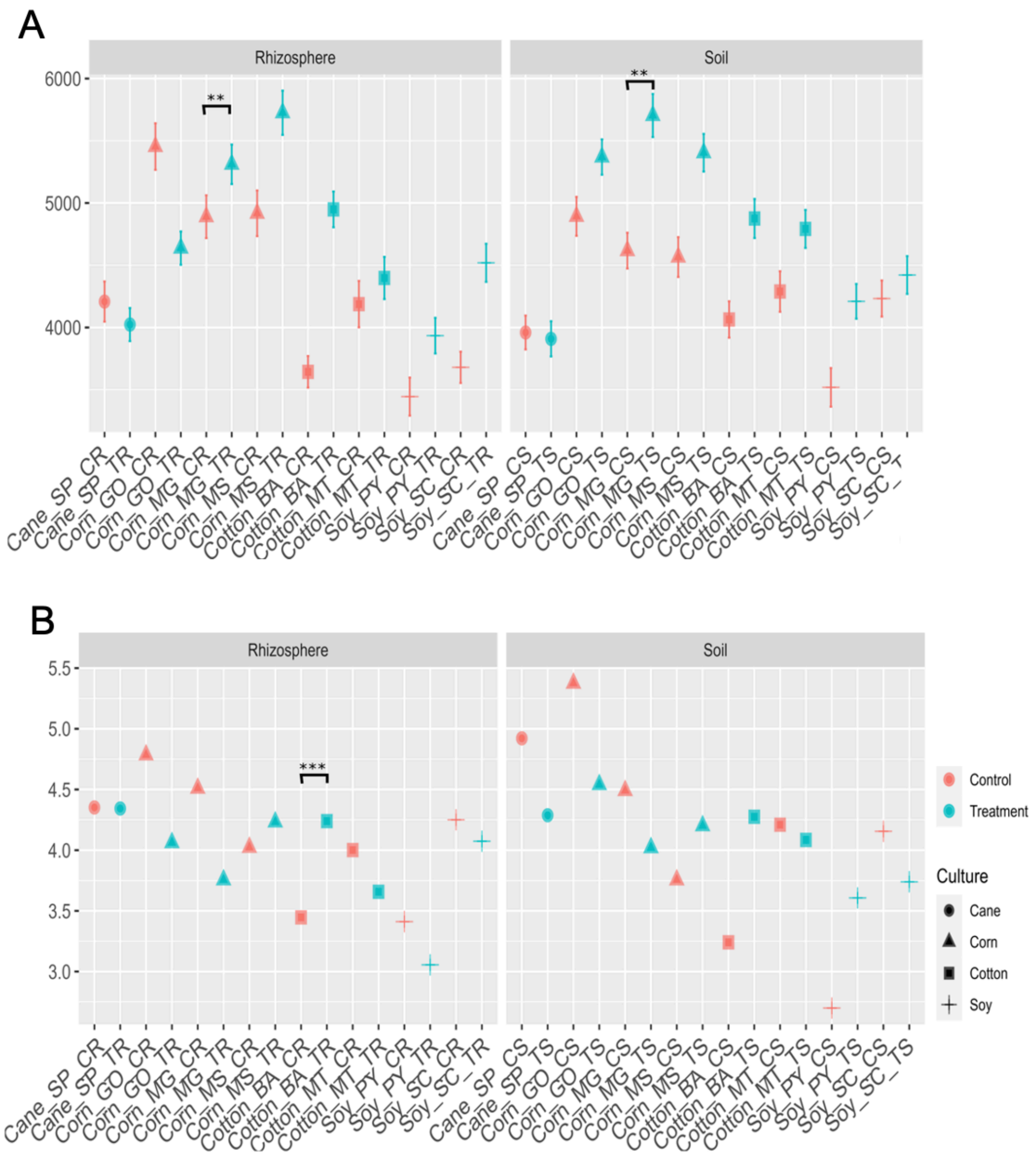


**Supplementary Figure 2.** Alpha diversity analysis of fungal communities using Chao1 (A) and Shannon (B) indices. The microbiome was analyzed based on their ITS amplicon sequencing. The lines in the graph represent the mean values of Chao1 and Shannon indices for each sample considering 3 replicates. Error bars represent standard deviations. Each crop is represented by a different color as indicated in the key. Locations and all four treatments are indicated, control (CR) or treatment (TR)). Significant differences are indicated by ** (pvalue< 0.05) or *** (pvalue< 0,01) according to T test.

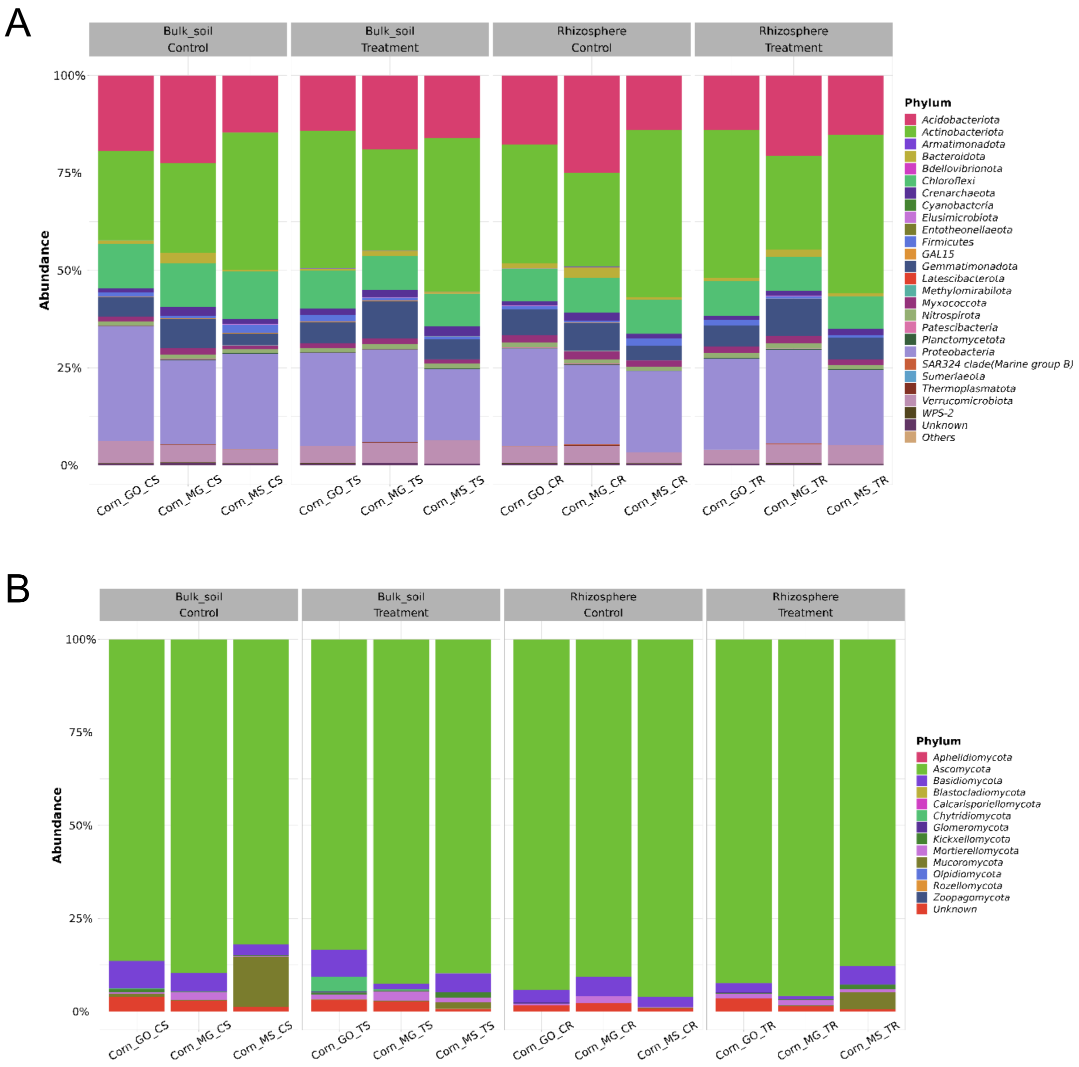


**Supplementary Figure 3.** Relative abundance of bacterial (A) and fungal (B) phyla in the microbiome associated with corn production systems in GO, MG and MS. Each bar represents one of the four treatments (average of 3 replicates) per location, i.e., control rhizosphere (CR), control soil (CS), treatment rhizosphere (TR) and treatment soil (TS). The color-coding in the bars corresponds to different phyla, as indicated in the key.

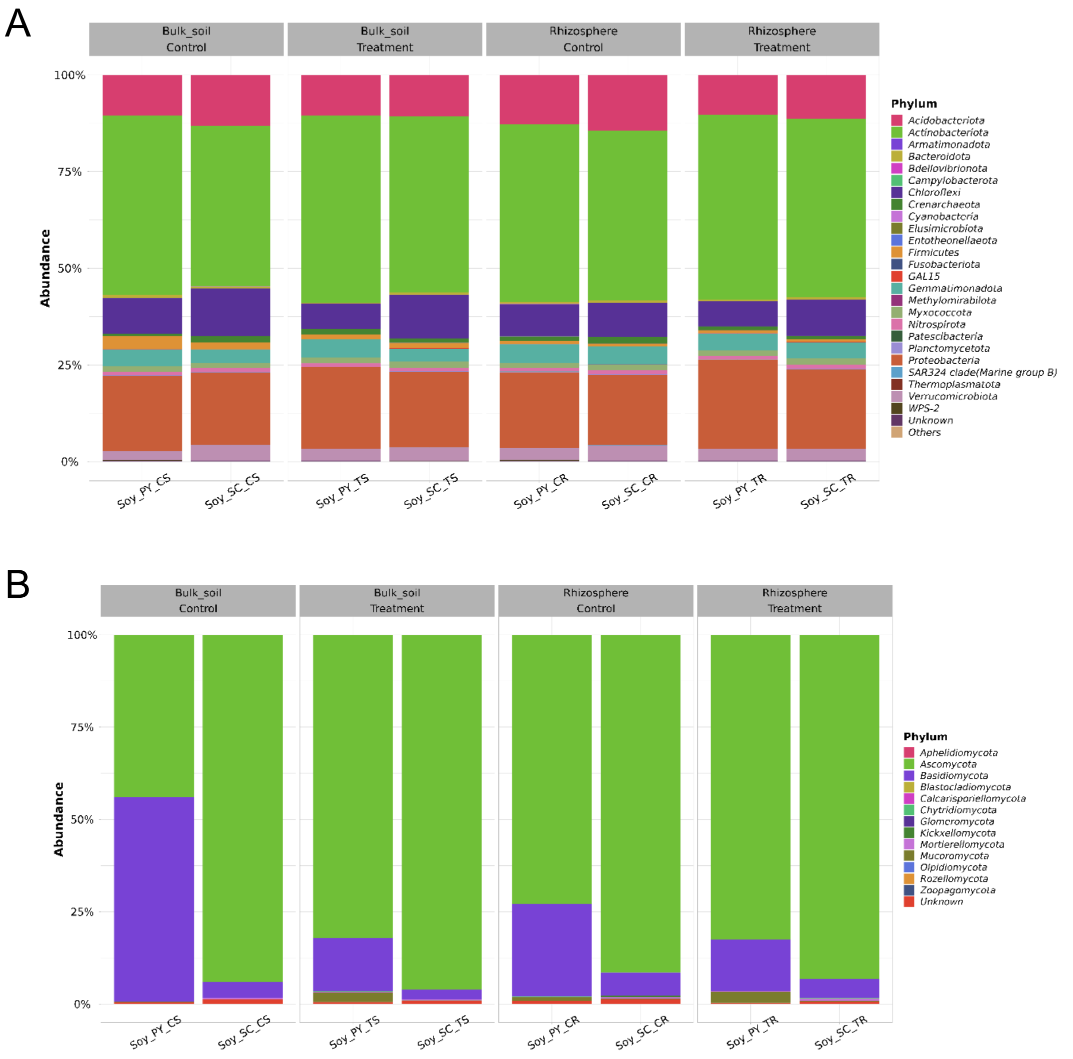


**Supplementary Figure 4.** Relative abundance of bacterial (A) and fungal (B) phyla in the microbiome associated with soy bean production systems in SC and PY. Each bar represents one of the four treatments (average of 3 replicates) per location, i.e., control rhizosphere (CR), control soil (CS), treatment rhizosphere (TR) and treatment soil (TS). The color-coding in the bars corresponds to different phyla, as indicated in the key.

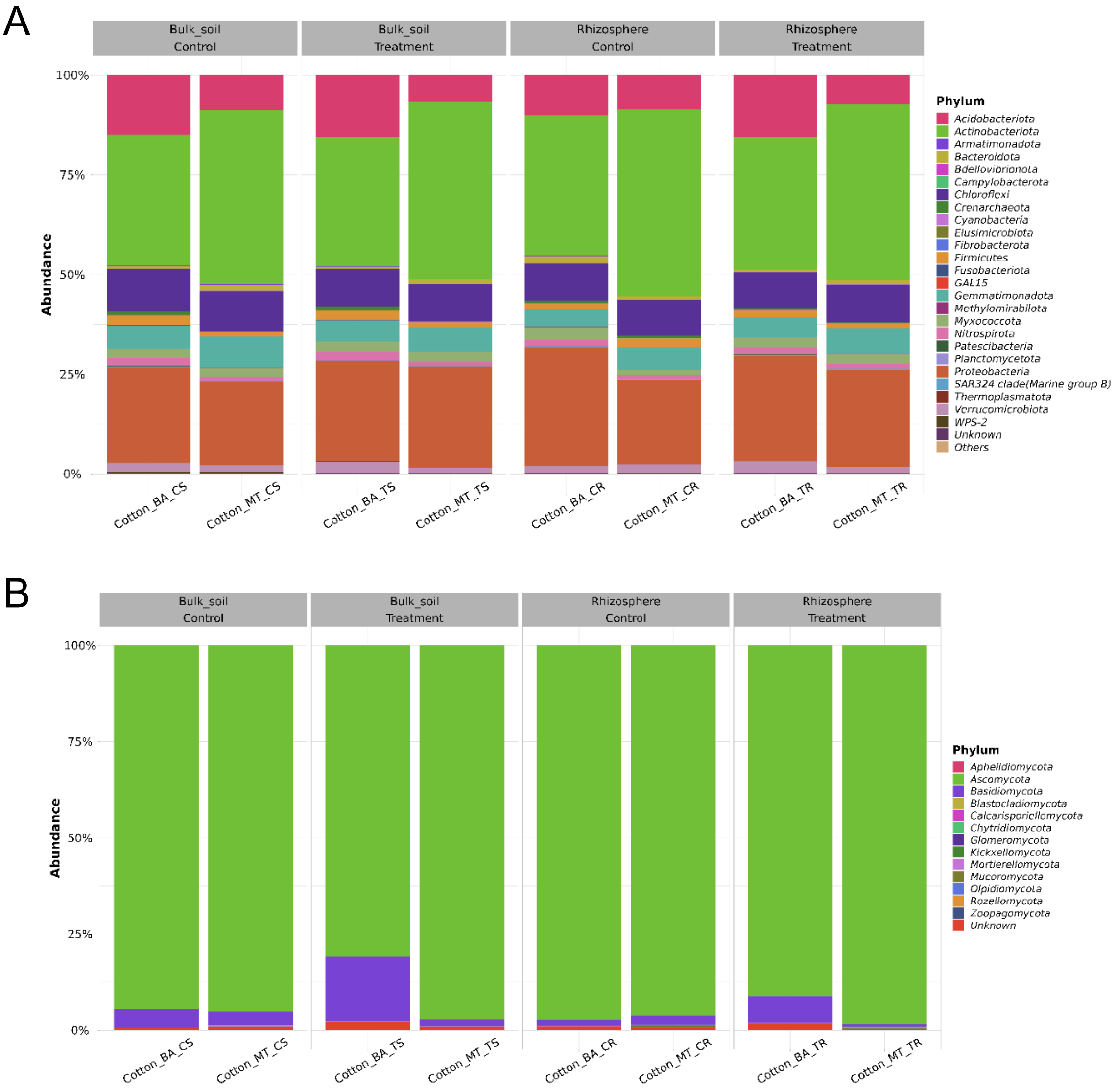


**Supplementary Figure 5.** Relative abundance of bacterial (A) and fungal (B) phyla in the microbiome associated with cotton production systems in MT and BA. Each bar represents one of the four treatments (average of 3 replicates) per location, i.e., control rhizosphere (CR), control soil (CS), treatment rhizosphere (TR) and treatment soil (TS). The color-coding in the bars corresponds to different phyla, as indicated in the key.

A

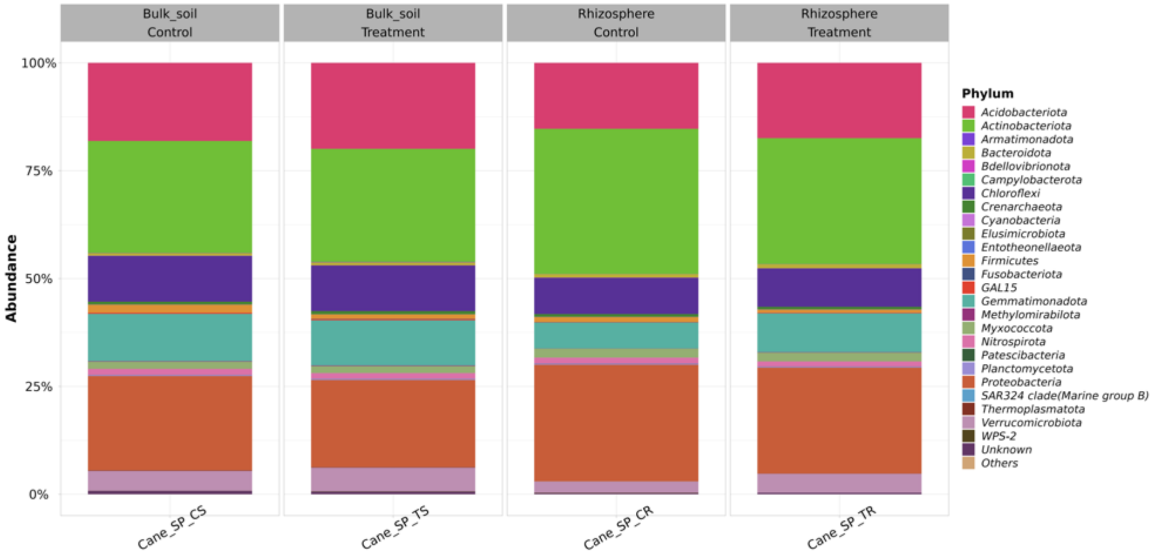


B

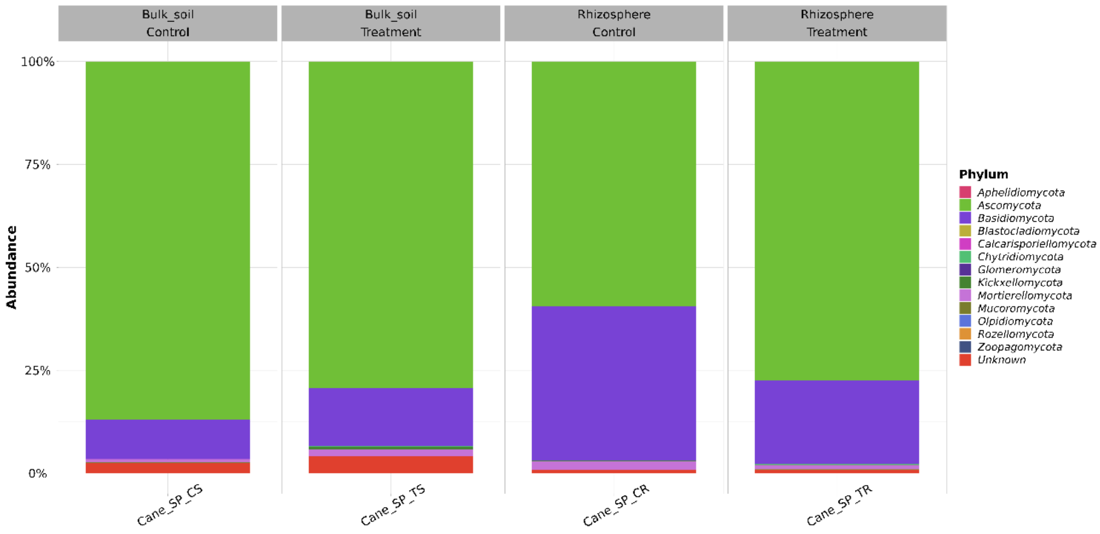


**Supplementary Figure 6.** Relative abundance of bacterial (A) and fungal (B) phyla in the microbiome associated with sugar cane production systems in SP. Each bar represents one of the four treatments (average of 3 replicates) per location, i.e., control rhizosphere (CR), control soil (CS), treatment rhizosphere (TR) and treatment soil (TS). The color-coding in the bars corresponds to different phyla, as indicated in the key.

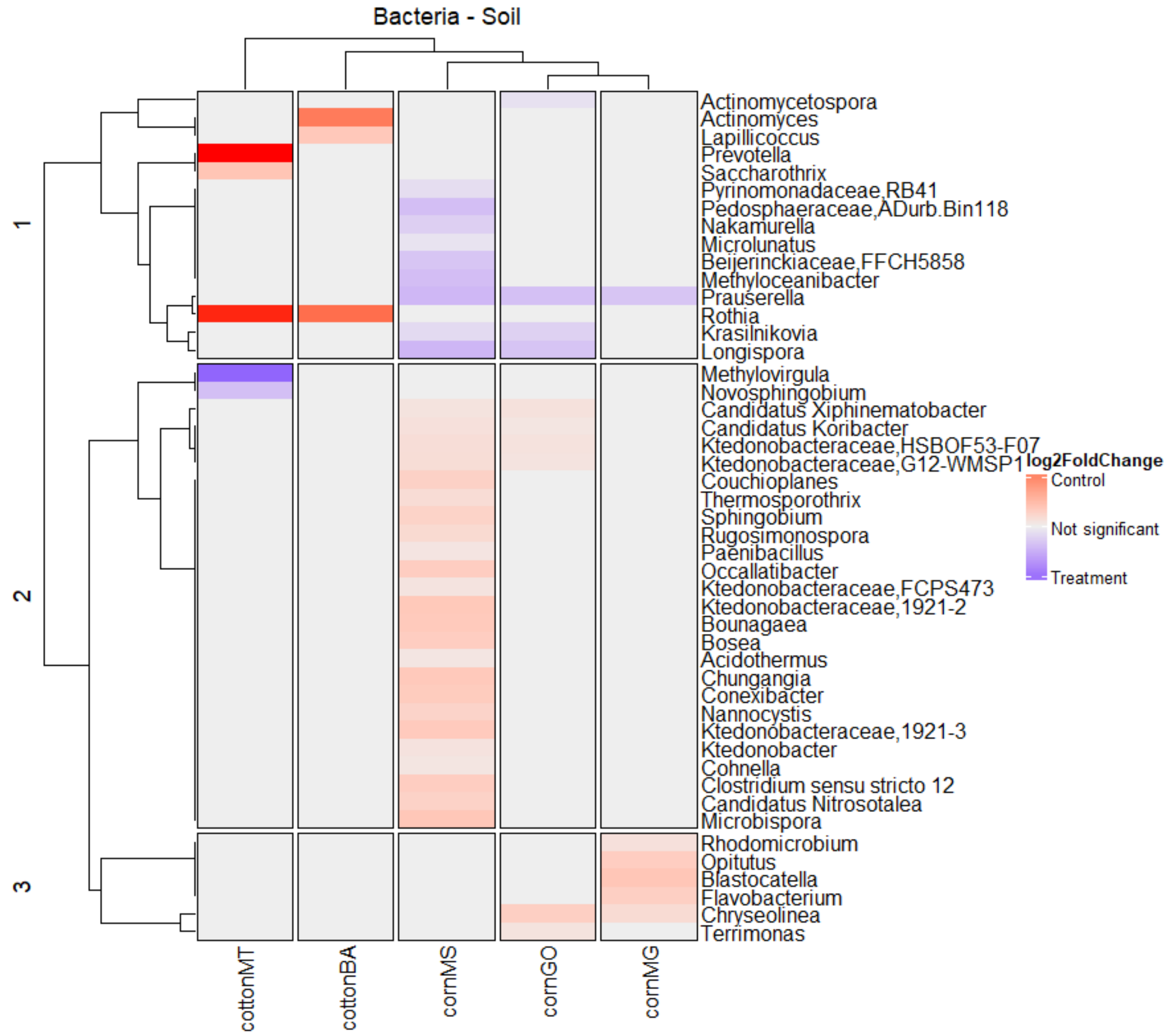


**Supplementary Figure 7.** Heatmap displaying changes in abundance of soil bacterial taxa significantly (p-value <0.05) enriched in control (red) or in the biostimulant (blue) treatment. The intensity of the color represents the magnitude of the log2 fold change. Only treatments with significant differences between treatments are show in the figure.

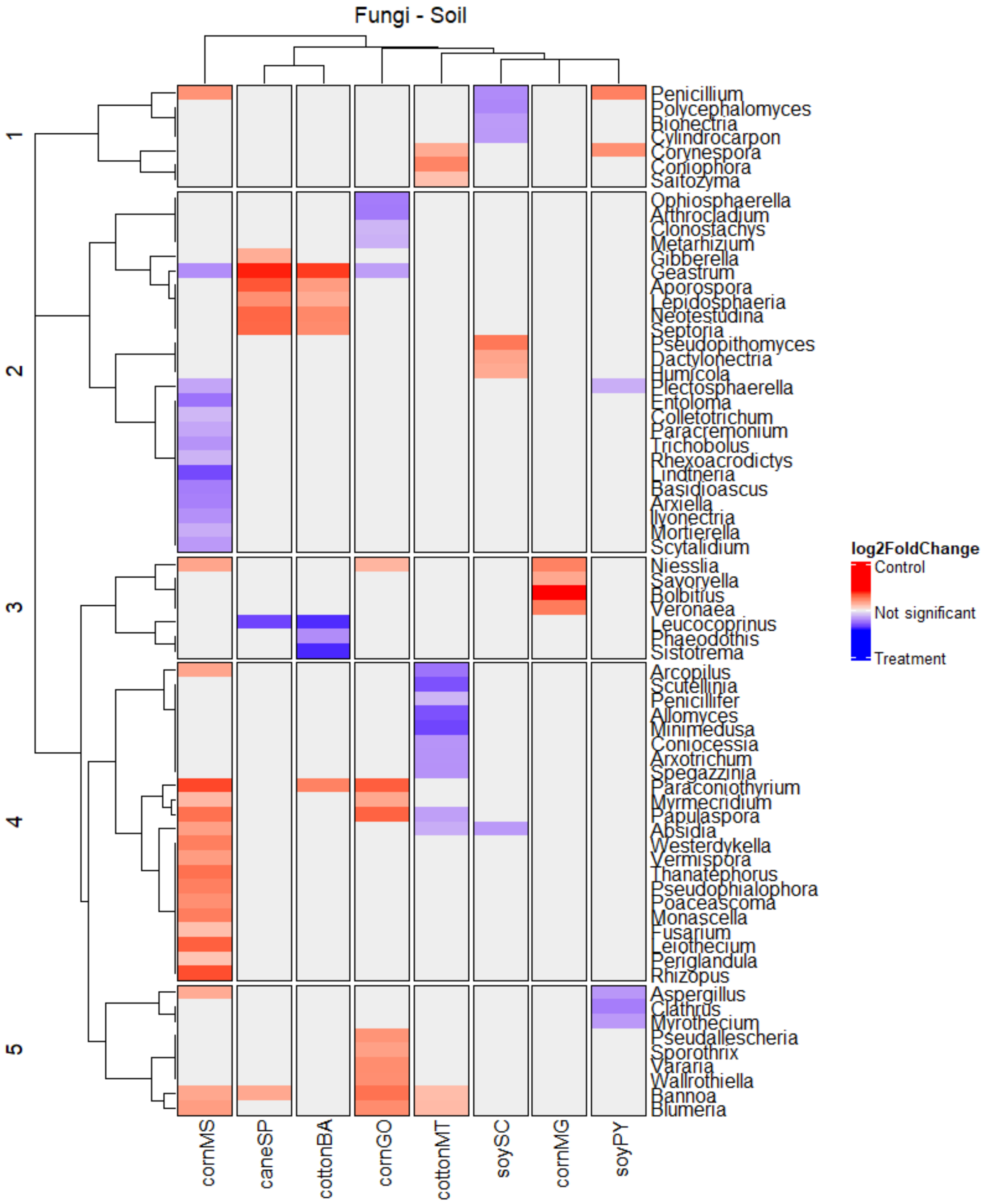


**Supplementary Figure 8.** Heatmap displaying changes in abundance of soil fungal taxa significantly (p-value <0.05) enriched in control (red) or in the biostimulant (blue) treatment. The intensity of the color represents the magnitude of the log2 fold change. Only treatments with significant differences between treatments are show in the figure.

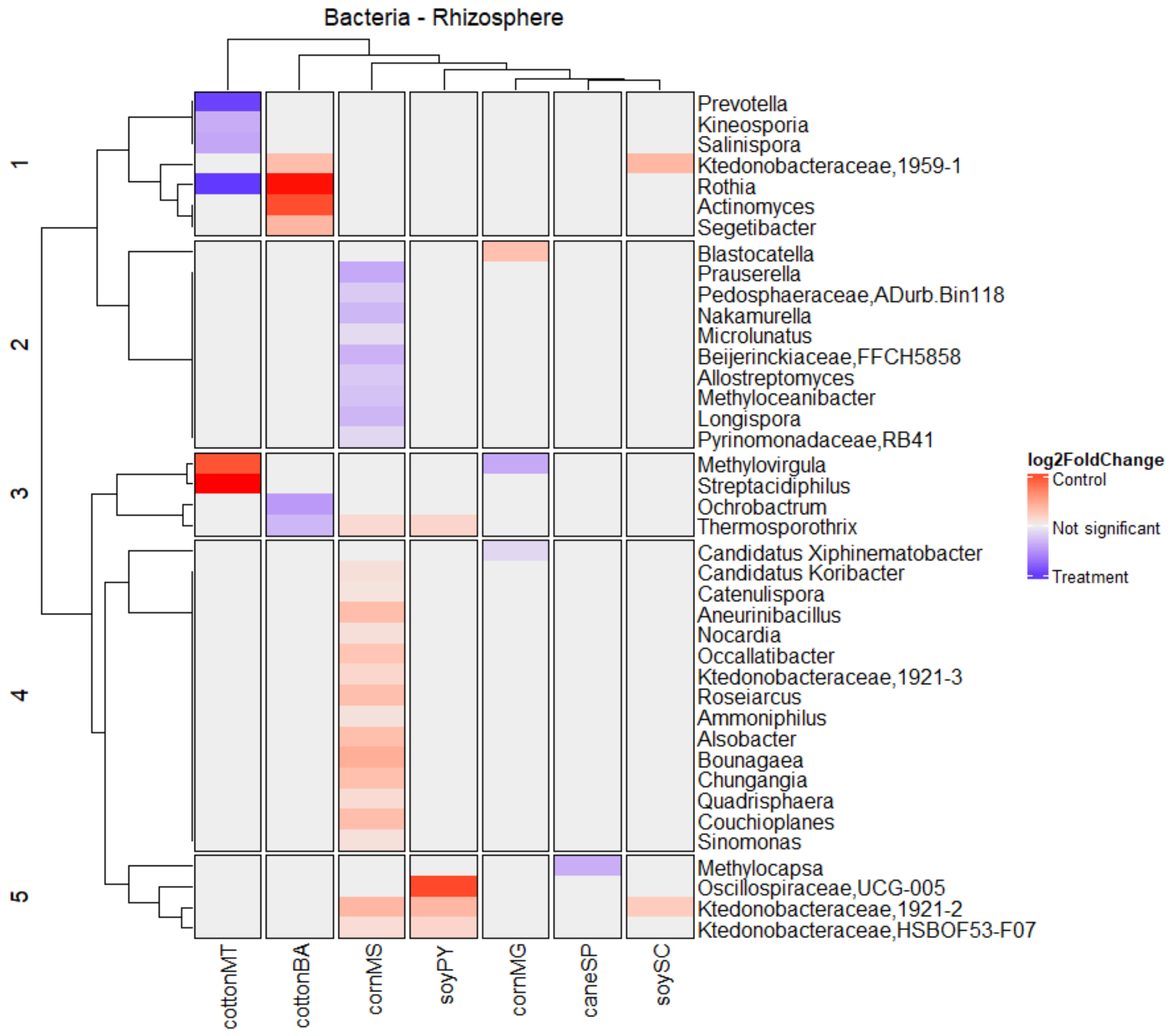


**Supplementary Figure 9.** Heatmap displaying changes in abundance of rhizosphere bacterial taxa significantly (p-value <0.05) enriched in control (red) or in the biostimulant (blue) treatment. The intensity of the color represents the magnitude of the log2 fold change. Only treatments with significant differences between treatments are show in the figure.

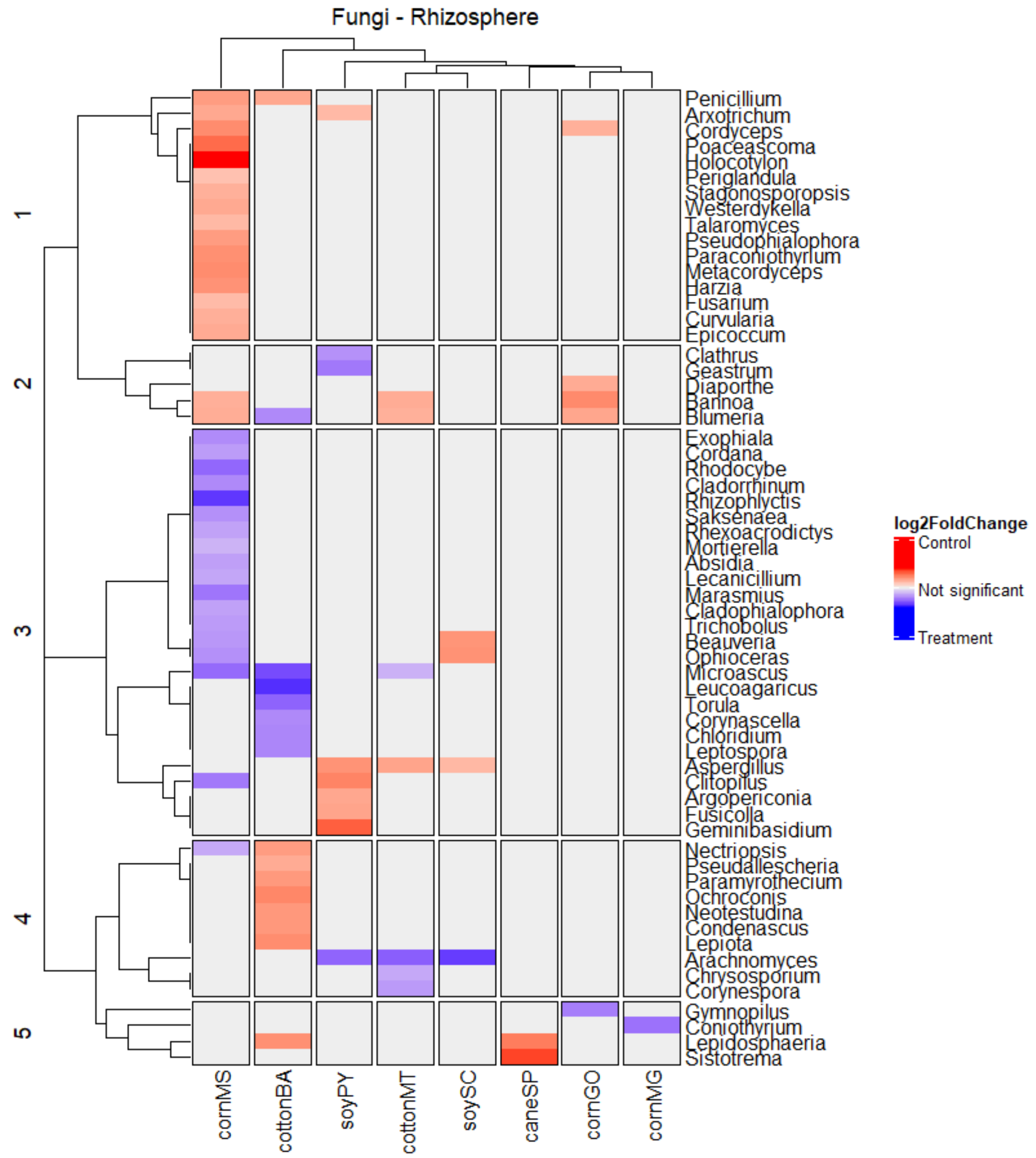


**Supplementary Figure 10.** Heatmap displaying changes in abundance of rhizosphere fungal taxa significantly (p-value <0.05) enriched in control (red) or in the biostimulant (blue) treatment. The intensity of the color represents the magnitude of the log2 fold change. Only treatments with significant differences between treatments are show in the figure.

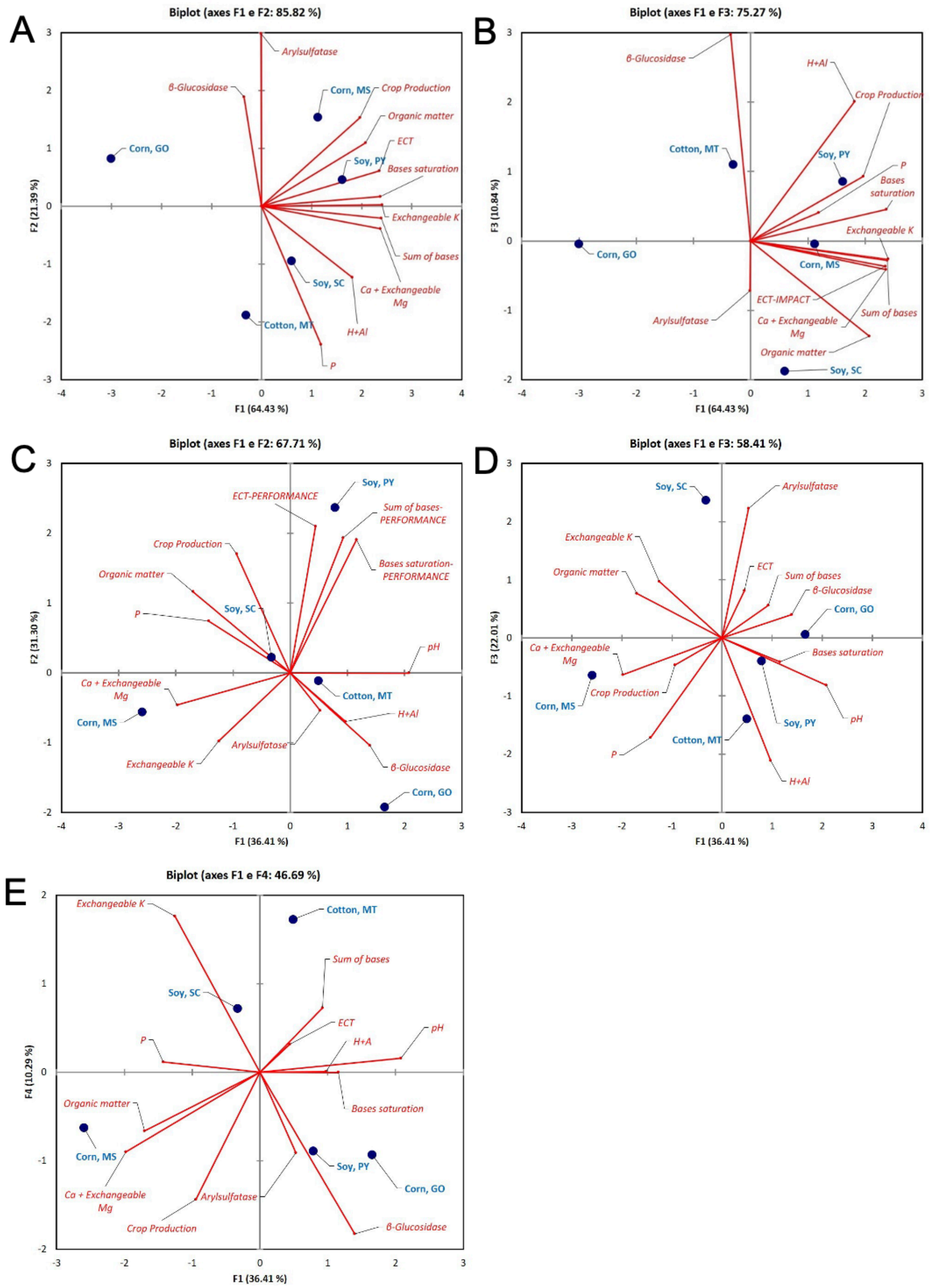


**Supplementary Figure 11.** Biplots of the Principal Components Analysis for impact values (A and B) and for technical performance (C, D and E) of biostimulant use showing the correlation circle of the variables (indicators, red vectors) and the observations cloud (case studies, blue dots) with normalized values.
